# Immunotherapy in thymic epithelial tumors: tissue predictive biomarkers for immune checkpoint inhibitors

**DOI:** 10.37349/etat.2024.00229

**Published:** 2024-05-21

**Authors:** Stefano Lucà, Marina Accardo, Severo Campione, Renato Franco

**Affiliations:** University of Rome La Sapienza, Italy; ^1^Pathology Unit, Department of Mental and Physical Health and Preventive Medicine, Università degli Studi della Campania “L. Vanvitelli”, 80138 Naples, Italy; ^2^Department of Advanced Diagnostic-Therapeutic Technologies and Health Services Section of Anatomic Pathology, A. Cardarelli Hospital, 80131 Naples, Italy

**Keywords:** Thymic epithelial tumors, immunotherapy, thymoma, thymic carcinoma

## Abstract

Thymic epithelial tumors (TETs) are rare malignant neoplasms arising in the thymus gland. Nevertheless, TETs, including thymomas (TMs), thymic carcinomas (TCs), and thymic neuroendocrine neoplasms (TNENs), are the most common mediastinal malignancies overall. A multidisciplinary approach is required for the appropriate diagnostic and therapeutic management of TETs. To date, the main therapeutic strategies are largely depended on the stage of the tumor and they include surgery with or without neoadjuvant or adjuvant therapy, represented by platinum-based chemotherapy, radiotherapy or chemoradiotherapy. Immune checkpoint inhibitors (ICIs) are ongoing under evaluation in the advanced or metastatic diseases despite the challenges related to the very low tumor mutation burden (TMB) and the high incidence of immune-related adverse events in TETs. In this regard, predictive impact of tissue biomarkers expression such as programmed cell death ligand-1 (PD-L1), and other emerging biomarkers, as well as their optimal and shared interpretation are currently under evaluation in order to predict response rates to ICIs in TETs.

## Introduction

Thymic epithelial tumors (TETs) are rare thoracic neoplasms arising from epithelial cells of the thymus gland [1]. Although less common than other thoracic neoplasms, such as pulmonary and pleural neoplasms [2–4]. TETs are the most frequently encountered tumors in the prevascular mediastinum [5]. TETs are a basket of different tumors with different clinical, histopathological, immunophenotypical, molecular and biological features at the base of clinical-pathological differences [6–9]. TETs share the lowest tumor mutation burden (TMB) among adult solid tumors [7, 10] and they, particularly thymomas (TMs), are often associated with peculiar autoimmune diseases, particularly myasthenia gravis, pure red cell aplasia and Good’s syndrome [11]. The choice of the optimal therapeutic treatment of TETs is primarily based on staging and histotype; in detail, surgery is the treatment of choice, being the only curative strategy in localized diseases, with eventually combined radiotherapy and/or chemotherapy on the base of surgical radicality, histotype and stage disease, while platinum-based chemotherapy is the standard of care for locally-advanced or metastatic TETs [12, 13]. However, therapeutic strategies for relapsed or refractory TETs are limited with different targeted agents (epidermal growth factor receptor inhibitors, inhibitors of angiogenesis, c-kit inhibitors, histone deacetylase inhibitors), since no real benefit has been shown in these clinical settings [14–17]. These data and the low TMB of tumors, limiting the identification of new therapeutic targets, explain the need to research new therapeutic strategies. In this regard, immune checkpoint inhibitors (ICIs) are a promising option just likes for other advanced stage malignant neoplasms [18–21].

## World Health Organization 2021 classification of TETs: a short summary

**Figure 1:**
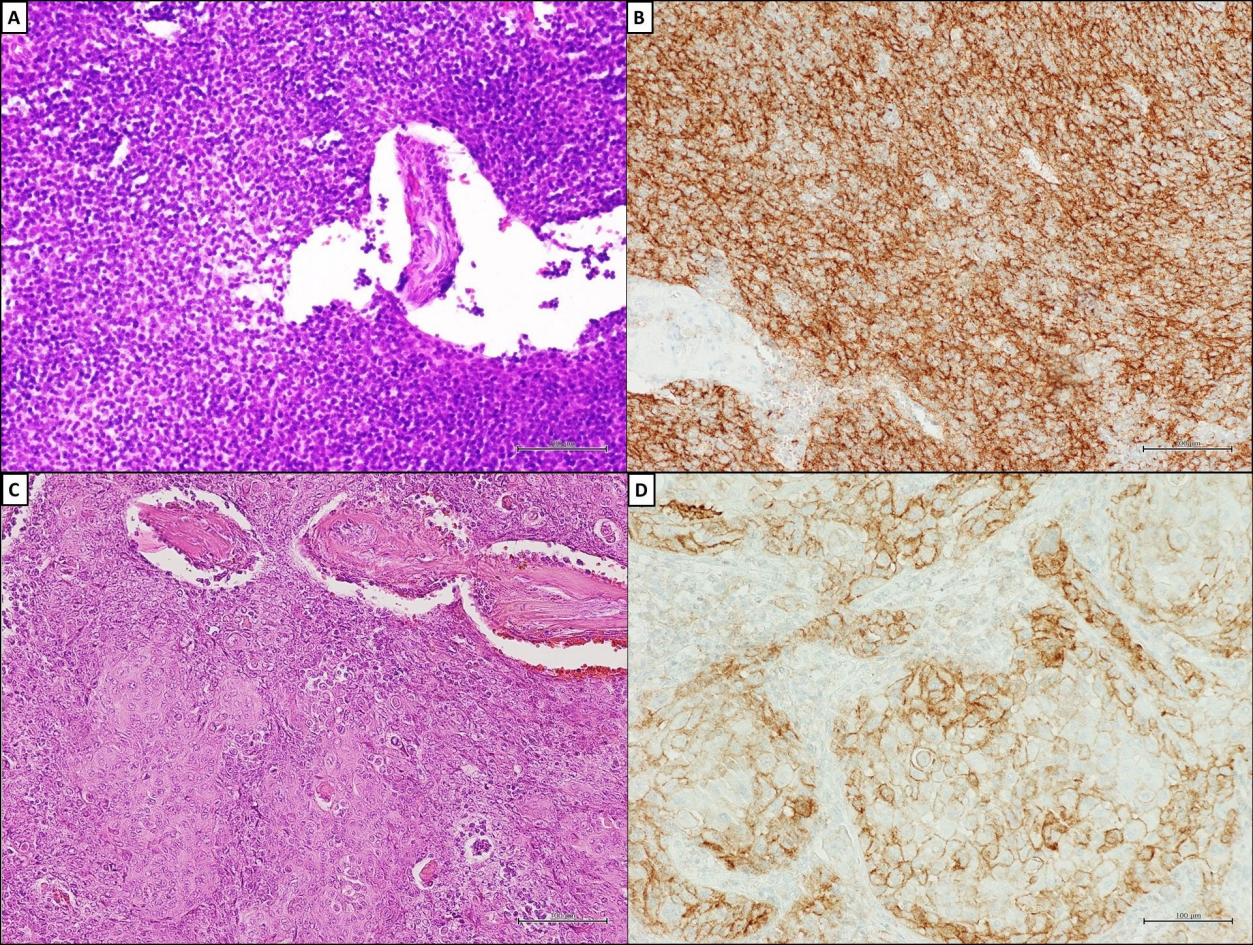
Programmed cell death ligand-1 (PD-L1) expression on tumor cells of TETs. (A) Histopathological appearance of type B2 TM; (B) diffuse and strong immunohistochemical (IHC) membranous staining (MS) for PD-L1 (clone SP263; Ventana Medical Systems) in ≥ 50% of neoplastic cells in type B2 TM and squamous cell carcinoma of the thymus; (C) squamous cell carcinoma of the thymus (hematoxylin and eosin); (D) squamous cell carcinoma of the thymus. A–D, scale bar = 100 μm; original magnifications ×200. Courtesy of Pathology Unit of Università degli Studi della Campania “L. Vanvitelli”

TETs arising from thymic epithelial cells, particularly thymic cortical epithelial cells (cTECs) or thymic medullary epithelial cells (mTECs) include TMs, thymic carcinomas (TCs) and thymic neuroendocrine neoplasms (TNENs) (Table 1) [6, 22]. TMs are the most common neoplastic type accounting for more than 50% of TETs, while TCs and TNENs represent approximately 14–22% and 2–5%, respectively [23]. TMs are characterized by thymus-like differentiation as they variably show organotypical features such as lobulated architecture, perivascular spaces, medullary differentiation and intratumoral infiltration of immature T-lymphocytes while Hassall corpuscles are only occasionally identified [6, 24, 25]. TMs are a heterogeneous group of neoplasm with different molecular, histopathological, immunophetypical and clinical features [26]. TMs are variably encapsulated and well circumscribed masses, except for type B3 TM which shows smooth invasive fronts with invasion in mediastinal fat or adjacent organs; neoplastic proliferation shows lobulated architecture due to the presence of thick fibrous septa and it is organized according to several growth patterns, with bland and spindle/oval cytomorphology and few or no admixed immature T-cells in type A TM, except for s atypical type A TM, and variably atypical polygonal neoplastic cells, as single cellular elements or arranged in clusters (≥ 3 contiguous cells), with dense immature T-lymphocyte infiltrate or few and scattered immature T lymphocytes in type B TMs (B1–B3). Furthermore, some TMs, such as metaplastic TM or micronodular TM with lymphoid stroma, show histopathological features that do not fit well with the most common histotypes and so they are classified separately. Finally, more than one histological subtype defines mixed cases of TM and the diagnosis should list the predominant pattern followed by any minor components in 10% increments, except for type AB TM which is considered a distinct TM subtype (Figure 1) [27–29]. TC is a very rare mediastinal tumor characterized by morphological features of obvious malignant biological behavior and with peculiar immunophenotypic expression of *CD5* and *CD117* (c-kit), unlike conventional carcinomas of other anatomical sites. Several histological types of TC are recognized (Figure 1) [6, 30]. TNENs are very rare thymic neoplasm accounting for 2–5% of all thymic tumors and they are currently classified just like their pulmonary counterpart, with the same nomenclature and the same diagnostic criteria (mitotic index/2 mm^2^, presence/absence of necrosis and cytomorphological features) of lung neutrophil extracellular traps (NETs) and neuroendocrine carcinoma (NECs) [31].

**Table 1:** World Health Organization (WHO) classification of TETs, 5th edition [6]

**TM**	**TC**	**TNENs**
Type A TM	Squamous cell carcinoma of the thymus	Typical carcinoid of the thymus
Type AB TM	Basaloid carcinoma of the thymus	Atypical carcinoid of the thymus
Type B1 TM	Lymphoepithelial carcinoma of the thymus	Small cell carcinoma of the thymus
Type B2 TM	Clear cell carcinoma of the thymus	Large cell neuroendocrine carcinoma of the thymus
Type B3 TM	Low-grade papillary adenocarcinoma of the thymus	-
Micronodular TM with lymphoid stroma	Mucoepidermoid carcinoma of the thymus	-
Metaplastic TM	TC with adenoid cystic carcinoma-like features	-
Lipofibroadenoma of the thymus	Enteric-type adenocarcinoma of the thymus	-
	Adenocarcinoma not otherwise specified (NOS) of the thymus	
	Adenosquamous carcinoma of the thymus	
	Sarcomatoid carcinoma of the thymus	
	Undifferentiated carcinoma of the thymus	
	TC NOS	

-: blank cells

## Immunotherapy in TETs

### Potential role of ICIs in TETs

Development of neoplasms is partially related to the inability of the immune system to eliminate neoplastic cells in the early stages of the disease [32]. Therefore, multiple immunotherapeutic strategies have been developed in order to activate and increase antitumor immunity [33]. ICIs are the most common form of immunotherapy strategy used in the clinical practice and they act by targeting programmed death 1 (PD-1)/PD-L1 axis or cytotoxic T-lymphocyte-associated protein 4 (CTLA-4) [34–37]. The efficacy of ICIs can be predicted by the evaluation of appropriate biomarkers of therapy response [38]. The level of PD-L1 expression in tumor cells and TMB are established as predictive biomarkers of therapeutic response in many solid tumors [19, 39, 40]. Recently, investigative efforts have been underway to identify predictive biomarkers of response to ICIs in TETs [41]. In this regard, TETs are characterized by the lowest TMB among all solid adult neoplasms [7, 10] and they are often associated with immune-mediated diseases [11]. These features are of great interest as they could negatively influence the use of ICIs in clinical practice. TMB reflects the number of non-synonymous single nucleotide variants (nsSNVs) in a neoplasm and therefore it is an expression of neo-antigens which can trigger an anti-tumor immune reaction, so determining the therapeutic response to ICIs; although a low TMB is associated with a low response rate to ICIs, the initial experience highlighted a surprisingly higher response rate to ICIs in TETs. The discordance between low TMB and a higher response rate is likely related to the specific biological function of the thymic gland and primarily to its role in T-cell development [42–45]. Moreover, the activation of antitumor immunity can also increase the risk of developing immune-related adverse events [46]. PD-L1 expression in TETs has been widely studied and overall data support its promising role as response predictor [8, 43, 44, 47–67]. Therefore, PD-L1 expression on tumor cells and its expression level appear to be the most promising biomarkers for immunotherapy response in TETs to date [41, 68, 69].

### PD-1/PD-L1 expression in TETs

PD-L1 expression on TETs tumor samples staining has been evaluated in many papers (Table 2) [8, 47–66] as well as its predictive value in patients with TETs who had progressed after at least one line of chemotherapy [43, 44, 70]. High PD-L1 and PD-1 IHC expression was widely observed in TETs. In detail, PD-L1 expression ranging from 23–92% of tumor cells in TMs and 36–100% of tumor cells in TCs [69]; variations in protein expression levels are related to various conditions, particularly disease stage (Masaoka stages) and subtype of TETs, with higher levels observed in thymic [70]. Rouquette et al. [67] compared the most used PD-L1 antibodies (i.e., clone E1L3N, clone 22C3, clone SP142, and clone SP263) in a cohort of 103 TETs (53 B3 TMs and 50 TCs) and observed a good concordance, using both 1% and 50% cut-off. However, no cut-off value for PD-L1 positivity has been uniformly and unequivocally recognized, to date. In this regard, it is interesting to report two clinical trials, which studied the effectiveness of ICIs in progressing after at one line of chemotherapy TETs [43, 44]. In their phase II clinical trial, Giaccone et al. [44] aimed to study the effectiveness of pembrolizumab and they used clone 22C3 in order to define PD-L1 expression of archival formalin-fixed paraffin-embedded tissues. They classified PD-L1 IHC expression as high, if at least 50% of the tumour cells stained positive; low if protein expression was observed in 1–49% of tumor cells and negative if no tumour cells in the sample expressed PD-L1. Progression free-survival (PFS) and overall survival (OS) were longer in patients with high PD-L1 expression than those with low or no expression, suggesting that PD-L1 expression in at least 50% of tumor cells correlated with a better response to ICIs (Table 3). Cho et al. [43] likewise investigated PD-L1 expression by immunohistochemistry using the same antibody (clone 22C3) and a similar interpretation protocol (PD-L1) positivity was defined by membranous PD-L1 staining in ≥ 1% of tumor and associated inflammatory cells or positive staining of stroma; PD-L1 expression was classified as high if at least 50% of the tumor cells, inflammatory cells, or stroma cells stained positive while PD-L1 expression in 0% to 49% of cells was classified as low expression). The trial results demonstrated that high PD-L1 immunohistochemistry expression was significantly associated with better response to pembrolizumab in TETs; indeed, overall response rate (ORR) was 35.7% in patients with PD-L1 expression levels higher than 50% (Table 3). Overall data suggest that ICIs (pembrolizumab) yielded encouraging antitumor activity with durable time in refractory, metastatic or relapsed TETs and that the best response rates are obtained with a PD-L1 expression greater than 50%, reporting a significant correlation between high PD-L1 levels and better and more durable response to ICIs.

**Table 2:** PD-L1 expression on TETs

**Reference**	**TM**	**TC**	**Antibody of PD-L1**	**IHC staining criteria of positivity**	**TM positivity rate**	**TC positivity rate**
Weissferdt et al. [47]	74/100 (74.00%)	26/100 (26.00%)	Clone EPR4877	MS > 5%	64.00%	54.00%
Arbour et al. [48]	12/23 (52.17%)	11/23 (47.83%)	Clone E1L3N	MS > 25%	92.00%	36.00%
Suster et al. [49]	-	21	Clone SP142	MS > 50%	NA	71.40%
Higuchi et al. [50]	31/39 (79.50%)	8/39 (20.50%)	Clone 28-8	MS ≥ 1%	51.60%	62.50%
Wei et al. [51]	100/169 (59.20%)	69/169 (40.80%)	Clone E1L3N	MS > 50%	36.00%	37.00%
Duan et al. [52]	20/33 (60.60%)	13/33 (39.40%)	Clone Ab58810	Intensity of staining score (1–3); median value of all scores as the cut-off value	65.00%	46.20%
Funaki et al. [53]	-	43	Clone SP142	MS > 50%	NA	60.50%
Katsuya et al. [54]	101/139 (72.60%)	38/139 (27.40%)	Clone E1L3N	H-score: score 3 [staining intensity (0–3) × % of positive cells (0–100%)]	23.00%	70.00%
Padda et al. [55]	65/69 (94.20%)	4/69 (5.80%)	Clone 15	Score 3 (intensity of MS 0–3)	68.00%	75.00%
Marchevsky et al. [56]	38/46 (82.60%)	8/46 (17.40%)	Clone SP142	MS ≥ 6%	92.00%	50.00%
Enkner et al. [8]	37/72 (51.30%)	35/72 (48.70%)	Clone E1L3N	H-score (cut-off value NA)	89.00%	53.00%
Katsuya et al. [57]	12/30 (40.00%)	18/30 (60.00%)	Clone E1L3N	Score ≥ 1 (intensity of MS 0–3)	67.00%	41.00%
Yokoyama et al. [58]	82	-	Clone EPR1161	Youden’s index > 38%	53.70%	NA
Tiseo et al. [59]	87/107 (81.30%)	20/107 (18.70%)	Clone E1L3N	H-score: score 3 (intensity of MS 0–3)	18.00%	65.00%
Owen et al. [60]	32/35 (91.40%)	3/35 (8.60%)	Clone 22C3	Score 1 (intensity of MS 0–5)	81.00%	100.00%
Hakiri et al. [61]	81	-	Clone SP142	MS ≥ 1%	27.00%	NA
Guleria et al. [62]	84	-	Clone SP263	MS > 25%	82.00%	NA
Chen et al. [63]	40/70 (57.00%)	30/70 (43.00%)	Clone SP142	MS ≥ 5%	37.50%	76.70%
Bagir et al. [64]	38/44 (86.30%)	6/44 (13.70%)	Clone AM26531AF-N	MS > 5%	81.60%	83.30%
Ishihara et al. [65]	55/66 (83.30%)	11/66 (16.70%)	Clone SP263	MS > 25%	92.70%	72.70%
Berardi et al. [66]	63/68 (92.60%)	5/68 (7.40%)	Clone 28-8	MS > 1%	Overall: 25.00%	

NA: not available; -: blank cells

**Table 3:** Completed clinical trials with ICIs in TETs

**Reference**	**Treatment**	**TM/TC**	**PD-L1 cut-off**	**PD-L1 positive cases**	**Primary endpoint**	**mOS**	**mPFS**	**ORR**
Giaccone et al. [44]	Pembrolizumab	0/40	PD-L1^high^: ≥ 50%	10/37 (27%)	ORR	Un	4.2	-
			PD-L1^low^: 1–49%	27/37 (73%)		15.5	2.9	
Cho et al. [43]	Pembrolizumab	7/26	PD-L1^high^: ≥ 50%	14/24 (58.3%)	ORR	-	-	35.7%
			PD-L1^low^: 1–49%	10/24 (41.7%)		-	-	NR

mPFS: median PFS; mOS: median OS; Un: unachieved; NR: no response; -: blank cells

### Emerging biomarkers for immunotherapy in TETs

Inhibition of the PD-1/PD-L1 axis is the most promising and most studied immunotherapeutic strategy in TETs. However, there are other immune checkpoints that could be targeted, such as, B7-H3, B7-H4, T cell immunoglobulin and mucin domain-containing protein 3 (TIM-3), and several co-stimulatory molecules, such as CD137, glucocorticoid-induced tumour necrosis factor receptor family-related protein (GITR), inducible co-stimulator (ICOS), regulating T-cell mediated anti-tumor response [36, 48, 71]. In detail, B7-H4 protein belongs to the B7 family. B7-H4 is a negative co-stimulatory molecule and allows tumor cells to escape immune surveillance; it also plays an essential role in the formation of the tumor microenvironment. B7-H4 protein usually has low expression in normal tissues but higher expression several solid neoplasms [72–74]. High expression of B7-H4 protein by IHC staining (anti-B7-H4 monoclonal antibody, clone EP1165) is positively correlated with high regulatory T cells and forkhead box protein P3 (FOXP3) expression in the microenvironment, thus it can indicate the suppressive immune microenvironment and this relation could predict poor prognosis in patients with TETs [71, 75]. The expression of tissue biomarkers such as TIM-3, CD137, GITR, ICOS and CTLA-4 on tumor infiltrating lymphocytes (TILs) of TETs has been recently evaluated. Arbour et al. [48] observed an expression of TIM-3 and GITR in all evaluated TETs samples while ICOS and CTLA-4 were positive in almost all the samples (91%), with a moderate to high expression of these biomarkers. These data suggest a synergistic action of anti-TIM-3 or CD137 agonist with anti-PD-1/PD-L1 blockade, highlighting the potential need to evaluate the tissue expression of these biomarkers [48]. Moreover, recent results have been shown a Wilms’ tumour 1 (WT1) IHC expression on tumor cells, underlining the value of WT1 peptide as an immunotherapy target, particularly WT1 peptide vaccination as a new avenue for treatment of advanced or recurrent TETs [76]. The *WT1* gene is a tumor suppressor and it’s overexpressed in several solid and non-solid neoplasms [77–83]. WT1 protein plays several oncogenic roles including involvement in cancer cell growth [84], resistance to apoptosis [85], enhancement of cell migration [86] and tumor vascularization [87]. WT1-targeting immunotherapeutic strategies have been developed in the past years [88–91]. Oji et al. [92] conducted the first phase II clinical trial of WT1 peptide vaccine immunotherapy for advanced TETs. In their report WT1 expression was assessed with IHC staining using a monoclonal anti-WT1 antibody (clone 6f-H2) and samples were scored as positive (WT1 overexpression) when more than 10% of tumor cells were stained in either their cytoplasm or nucleus. Their interesting results showed as WT1 was overexpressed in the majority of TETs (11 of 13 TCs and 4 of 5 TMs) and that vaccination with WT1 peptide induced WT1-specific immune responses and WT1 peptide immunotherapy had clinical potential with a stable disease rate of 75.0% both in patients with TCs and TMs at 3 months. Thus, although the data is still limited, WT1 overexpression in TETs provides an opportunity to develop specific cancer vaccines [93]; however, the impact of this therapeutic strategy on the development of autoimmune-related complications is not yet known. Therefore, future clinical studies are needed to demonstrate the real therapeutic value of WT1 peptide-based immunotherapy and to study the association of WT1 peptide vaccine with the development of autoimmune-related complications in TETs [76, 94].

## Conclusions

Immunotherapy is currently not a standard-of-care in TETs but ICIs have demonstrated encouraging clinical activity in relapsed and refractory TETs, although their administration is associated with a high risk of developing or precipitating immune-related adverse events in this clinical setting. Despite the rarity of the tumors, many papers demonstrated significant expression levels of PD-L1 on TETs cells, both as a percentage of immunopositive tumor cells and as intensity of expression. Most of these papers have however evaluated only the percentage of staining cells to define positivity cut-offs, just like the completed clinical trials. In addition, the expression of co-inhibotory immune checkpoints and co-stimulatory molecules regulating antitumor response on TETs tissue samples has been evaluated. Taken together, all these data provide a rationale for using ICIs for treatment of TETs and defining a standardized, univocal and reproducible evaluation protocol for predictive tissue biomarkers, particularly PD-L1, in order to pave the way for the personalized use of ICIs inhibitors in TETs.

## Abbreviations

CTLA-4: cytotoxic T-lymphocyte-associated protein 4
GITR: glucocorticoid-induced tumour necrosis factor receptor family-related protein
ICIs: immune checkpoint inhibitors
ICOS: inducible co-stimulator
IHC: immunohistochemical
MS: membranous staining
ORR: overall response rate
PD-1: programmed death 1
PD-L1: programmed cell death ligand-1
TCs: thymic carcinomas
TETs: thymic epithelial tumors
TIM-3: T cell immunoglobulin and mucin domain-containing protein 3
TMB: tumor mutation burden
TMs: thymomas
TNENs: thymic neuroendocrine neoplasms
WT1: Wilms’ tumour 1

## Declarations

### Copyright

© The Author(s) 2024.
